# The Estimation of Cortical Activity for Brain-Computer Interface: Applications in a Domotic Context

**DOI:** 10.1155/2007/91651

**Published:** 2007-09-03

**Authors:** F. Babiloni, F. Cincotti, M. Marciani, S. Salinari, L. Astolfi, A. Tocci, F. Aloise, F. De Vico Fallani, S. Bufalari, D. Mattia

**Affiliations:** ^1^Istituto di Ricovero e Cura a Carattere Scientifico, Fondazione Santa Lucia, Via Ardeatina, 306-00179 Rome, Italy; ^2^Dipartimento di Fisiologia Umana e Farmacologia, Università di Roma “La Sapienza”, Piazzale Aldo Moro 5, 00185 Rome, Italy; ^3^Dipartimento di Informatica e Sistemistica, Università di Roma “La Sapienza”, Piazzale Aldo Moro 5, 00185 Rome, Italy

## Abstract

In order to analyze whether the use of the cortical activity, estimated from noninvasive EEG recordings, could be useful to detect mental states related to the imagination of limb movements, we estimate cortical activity from high-resolution EEG recordings in a group of healthy subjects by using realistic head models. Such cortical activity was estimated in region of interest associated with the subject's Brodmann areas by using a depth-weighted minimum norm technique. Results showed that the use of the cortical-estimated activity instead of the unprocessed EEG improves the recognition of the mental states associated to the limb movement imagination in the group of normal subjects. The BCI methodology presented here has been used in a group of disabled patients in order to give them a suitable control of several electronic devices disposed in a three-room environment devoted to the neurorehabilitation. Four of six patients were able to control several electronic devices in this domotic context with the BCI system.

## 1. INTRODUCTION

Brain-computer interface (BCI) is an area of research that is rapidly growing in the neuroscience and bioengineering fields. One popular approach to the generation of a BCI system consists in the recognition of the patterns of electrical activity on the scalp gathered from a series of electrodes by a computer. One of the problems related to the use of surface EEG is the blurring effect due to the smearing of the skull on the transmission of the potential distribution from the cerebral cortex toward the scalp electrodes. This happens since the skull has a very low electric conductivity when compared with the scalp or the brain one. The blurring effect makes the EEG data gathered from the scalp electrodes rather correlated, a problem not observed in the cortical EEG data recorded from the invasive implants in monkeys and man. Such correlation makes the work of the classifiers problematic, since the features extracted from the different scalp electrodes tend to be rather similar and this correlation is hard to be disentangled with blind methods like principal component analysis.

In this last decade, high-resolution EEG technologies have been developed to enhance the spatial information content of EEG activity [1, 2]. Furthermore, since the ultimate goal of any EEG recording is to provide useful information about the brain activity, a body of mathematical techniques, known as inverse procedures, has been developed to estimate the cortical activity from the raw EEG recordings. Examples of these inverse procedures are the dipole localization, the distributed source, and the cortical imaging techniques [1–4]. Inverse procedures could use linear and nonlinear techniques to localize putative cortical sources from EEG data by using mathematical models of the head as volume conductor.

More recently, it has been suggested that, with the use of the modern high-resolution EEG technologies, it could be possible to estimate the cortical activity associated with the mental imagery of the upper limbs movements in humans better than with the scalp electrodes [4–6]. We currently use this technology to estimate the cortical current density in particular region of interest (ROI) on the modeled brain structures from high-resolution EEG recordings to provide high-quality signals for the extraction of the features useful to be employed in a BCI system.

In this paper, we would like to illustrate how, with the use of such advanced high-resolution EEG methods for the estimation of the cortical activity, it is possible to run a BCI system able to drive and control several devices in a domotic environment. In particular, we first describe a BCI system used on a group of normal subjects in which the technology of the estimation of the cortical activity is illustrated. Then, we used the BCI system for the command of several electronic devices within a three-room environment employed for the neurorehabilitation. The BCI system was tested by a group of six patients.

## 2. METHODOLOGY

**Subjects**

Two groups of subjects have been involved in the training with the BCI system. One was composed of normal healthy subjects while the second one was composed of disabled persons who used the BCI system in attempt to drive electronic devices in a three-room facility at the laboratory of the foundation of Santa Lucia in Rome. The first group was composed by fourteen healthy subjects that voluntarily participated to the study. The second group of subjects were formed by six patients affected by Duchenne muscular dystrophy. According to the Barthel index (BI) score for their daily activity, all patients depended almost completely on caregivers, having a BI score lower than 35. In general, all patients were unable to walk since they were adolescent, and their mobility was possible only by a wheelchair which was electric in all (except two) patients and it was driven by a modified joystick which could be manipulated by either the residual “fine” movements of the first and second fingers or the residual movements of the wrist. As for the upper limbs, all patients had a residual muscular strength either of proximal or distal arm muscles that was insufficient for carrying on any everyday life activity. The neck muscles were as weak as to require a mechanical support to maintain the posture in all of them. Finally, eye movements were substantially preserved in all of them. At the moment of the study, none of the patients was using technologically advanced aids.

### 2.1. Patient’s preparation and training

Patients were admitted for a neurorehabilitation program that includes also the use of BCI system on a voluntary base. Caregivers and patients gave the informed consent for the recordings in agreement with the ethical committee rules adopted for this study. The rehabilitation programs aimed to allow to the patients the use of a versatile system for the control of several domestic devices by using different input devices, tailored on the disability level of the final user. One of the possible inputs for this system was the BCI by using the modulation of the EEG.

The first step of the clinical procedure consisted of an interview and a physical examination performed by the clinicians, wherein several levels of the variables of interest (and possible combinations) were addressed as follows: the degree of motor impairment and of reliance on the caregivers for everyday activities as assessed by current standardized scale, that is, the Barthel Index (BI) for ability to perform daily activities; the familiarity with transducers and aids (sip/puff, switches, speech recognition, joysticks) that could be used as the input to the system; the ability to speak or communicate, being understandable to an unfamiliar person; the level of informatics alphabetization measured by the number of hours per week spent in front of a computer. Information was structured in a questionnaire administered to the patients at the beginning and the end of the training. A level of system acceptance by the users was schematized by asking the users to indicate, with a number ranging from 0 (not satisfactory) to 5 (very satisfactory), their degree of acceptance relative to each of the controlled output devices. The training consisted of weekly sessions; for a period of time ranging from 3 to 4 weeks, the patient and (when required) her/his caregivers were practicing with the system. During the whole period, patients had the assistance of an engineer and a therapist in their interaction with the system.

### 2.2. Experimental task

Both normals and patients were trained by using the BCI system in order to control the movement of a cursor on the screen on the base of the modulation of their EEG activity. In particular, the description of the experimental task performed by all of them during the training follows. Each trial consisted of four phases.

1. Target appearance: a rectangular target appeared on the right side of the screen, covering either the upper or the lower half of the side.
2. Feedback phase: one second after the target, a cursor appeared in the middle of the left side of the screen and moved at a constant horizontal speed to the right. Vertical speed was determined by the amplitude of sensorimotor rhythms (see Section 2.6). A cursor sweep lasted about three seconds.
3. Reward phase: if the cursor succesfully hit the target, the latter flashed for about one second. Otherwise, it just disappeared.
4. Intertrial interval: the screen stayed blank for about two seconds, in which the subject was allowed to blink and swallow.

Subjects were aware that the increase or decrease of a specific rhythm in their EEG produces a movement of the cursor towards the top or the bottom of the screen. They were suggested to concentrate on kinesthetic imagination of upper limb movements (e.g., fist clenching) to produce a desynchronization of the rhythm on relevant channels (cursor up), and to concentrate on kinesthetic imagination of lower limb movements (e.g., repeated dorsiflexion of ankle joint) to produce a contrasting pattern (with possible desynchronization of *μ*/*β* rhythm over the mesial channels, cursor down). Using this simple binary task as performance measure, training is meant to improve performances from 50–70% to 80–100% of correct hits.

### 2.3. Experimental training

The BCI training was performed using the BCI2000 software system [7]. An initial screening session was used to define the ideal locations and frequencies of each subject's spontaneous *μ*- and *β*-rhythm activity. During this session, the subject was provided with any feedback (any representation of her/his *μ* rhythm), and she/he had to perform motor tasks just in an open loop. The screening session consisted in the alternate and random presentation of cues on opposite sides of the screen (either up/down -vertical- or left/right -horizontal). In two subsequent runs, the subject was asked to execute (first run) or to image (second run) movements of her/his hands or feet upon the appearance of top or bottom target, respectively. This sequence was repeated three times. From the seventh run on, the targets appeared on the left or right side of the screen, and the subject was asked to move (odd trials) or to image (even trials) her/his hands for a total of 12 trials. The offline analysis based on pairs of contrasts for each task aimed at detecting two, possibly independent, groups of features which will be used to train the subject to control two independent dimensions in the BCI. Analysis was carried on by replicating the same signal conditioning and feature extraction that was also used in the online processing (training session). Datasets are divided into epochs (usually 1-second long) and spectral analysis is performed by means of a maximum entropy algorithm, with a resolution of 2 Hz.

Different from the online processing, when the system only computes the few features relevant for BCI control, all possible features in a reasonable range are extracted and analyzed simultaneously. A feature vector is extracted from each epoch composed by the spectral value at each frequency bin between 0 and 60 Hz for each spatially filtered channel. When all features in the two datasets under contrast have been extracted, a statistical analysis is performed to assess significant differences in the values (epochs) of each feature in the two conditions. Usually an *r* ^2^ analysis is performed, but in the case of 2-level-independent variables (such in case tasks = {*T*1, *T*2}, t-test, ANOVA, etc.) would provide the analogous results. At the end of this process, the results were available (channel-frequency matrix and head topography of *r* ^2^ values) and evaluated to identify the most promising set of features to be enhanced with training.

Using information gathered from the offline analysis, the experimenter set the online feature extractor so that a “control signal” was generated from the linear combination of time-varying value of these features, and then passed to a linear classifier. The latter's output controls how the position of the feedback cursor was updated. During the following training sessions, the subjects were thus fed back with a representation of their *μ*-rhythm activity, so that they could learn how to improve its modulation.

Each session lasted about 40 minutes and consisted of eight 3-minute runs of 30 trials. The task was increased in difficulty during the training, so mainly two different task classes can be defined.

During the training sessions, subjects were asked to perform the same kinaesthetic imagination movement they were asked during the screening session. An upward movement of the cursor was associated to the bilateral decrease of *μ* rhythm over the hand area (which usually occurs during imagination of upper limb movement). Consequently, the (de)synchroinization pattern correlated to imagination of lower limb movements made the cursor move downwards. With the same principle, the horizontal movement of the cursor to the left (right) was linked to the lateralization of *μ* rhythm due to imagination of movement of the left (right).

To do so, two different control signals were defined. The vertical control signal was obtained as the sum of the *μ*-rhythm amplitude over both hand motor areas; the value of *μ*-rhythm amplitude over the foot area was possibly subtracted (depending on the individual subject's pattern). The horizontal control channel was obtained as the difference between the *μ*-rhythm amplitudes over each hand motor areas.

During the first 5–10 training sessions, the user is trained to optimize modulation of one control signal at a time, that is, overall amplitude (“vertical control”) or lateralization (“horizontal control”) of the *μ* rhythm. Either control channel was associated with vertical or horizontal movement of a cursor on the screen, respectively.

For the training of “vertical” control, the cursor moved horizontally across the screen from left to right at a fixed rate, while the user controlled vertical movements towards appearing targets, justified to the right side of the screen. Analogously, for the training of “horizontal” control, the cursor moved vertically across the screen from top to bottom at a fixed rate, while the user controlled horizontal movements towards appearing targets justified to the bottom side of the screen.

This phase was considered complete when the healthy subjects reached a performance of 70–80% correct hits (60–65% for patients) on both monodimensional tasks. In case of bidimensional task that was performed only by the normal subjects, the cursor appeared in the center, and its movement was entirely controlled by the subject, using both control channels (“horizontal” and “vertical”) simultaneously.

### 2.4. Domotic systemprototype features

The system core that disabled patients attempted to use in order to drive electronic devices in a three-room laboratory was implemented as follows. It received the logical signals from several input devices (including the BCI system) and converted them into commands that could be used to drive the output devices. Its operation was organized as a hierarchical structure of possible actions, whose relationship could be static or dynamic. In the static configuration, it behaved as a “cascaded menu” choice system and was used to feed the feedback module only with the options available at the moment (i.e., current menu). In the dynamic configuration, an intelligent agent tried to learn from the use which would have been the most probable choice the user will make. The user could select the commands and monitor the system behavior through a graphic interface. The prototype system allowed the user to operate remotely electric devices (e.g., TV, telephone, lights, motorized bed, alarm, and front door opener) as well as monitoring the environment with remotely controlled video cameras. While input and feedback signals were carried over a wireless communication, so that mobility of the patient was minimally affected, most of the actuation commands were carried via a powerline-based control system. As described above, the generated system admits the BCI as one possible way to communicate with it, being open to accept command by other signals related to the residual ability of the patient. However, in this study we report only the performance of these patients with the BCI system in the domotic applications.

### 2.5. Estimation of the cortical activity from the EEG recordings

**Figure 1:**
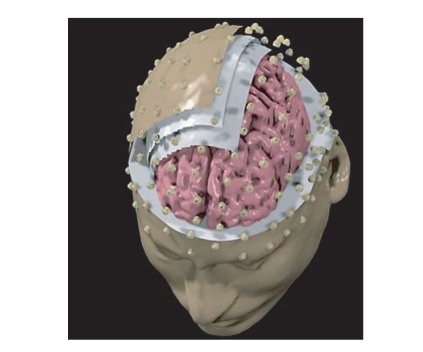
A realistic head model employed for the estimation of the cortical activity. Three layers are displayed, namely, representing dura mater, skull, and scalp. Also the electrode positions are visible on the scalp surface.

For all normal subjects analyzed in this study, sequential MR images were acquired and realistic head models were generated. For all the patients involved in this study, due to the lack of their MR images, we used the Montreal average head model. Figure 1 shows realistic head models generated for a particular experimental subjects, together with the employed high-resolution electrode array. Scalp, skull, dura mater, and cortical surfaces of the realistic and averaged head models were obtained. The surfaces of the realistic head models were then used to build the boundary element model of the head as volume conductor employed in the present study. Conductivities values for scalp, skull, and dura mater were those reported in Oostendorp et al. [8]. A cortical surface reconstruction was accomplished for each subject's head with a tessellation of about 5000 triangles on average, while the average head model has about 3000 triangles.

The estimation of cortical activity during the mental imagery task was performed in each subject by using the depth-weighted minimum norm algorithm [9, 10]. Such estimation returns a current density estimate for each one of the thousand dipoles constituting the modeled cortical source space. Each dipole returns a time-varying amplitude representing the brain activity of a restricted patch of cerebral cortex during the entire task time course. This rather large amount of data can be synthesized by computing the ensemble average of all the dipoles magnitudes belonging to the same cortical region of interest (ROI). Each ROI was defined on each subject's cortical model adopted in accordance with its Brodmann areas (BAs). Such areas are regions of the cerebral cortex whose neurons sharing the same anatomical (and often also functional) properties. Actually, such areas are largely used in neuroscience as a coordinate system for sharing cortical activation patterns found with different neuroimaging techniques. In the present study, the activity in the following ROI was taken into account: the primary left and right motor areas, related to the BA 4, the left and right primary somatosensory and supplementary motor areas.

### 2.6. Online processing

Digitized EEG data were transmitted in real time to the BCI2000 software system [7] which performed all necessary signal processing and displayed feedback to the user. The processing pipe can be considered of several stages, which process the signal in sequence. Only the main ones will be mentioned below: spatial filter, spectral feature extraction, feature combination, and normalization.

**Spatial filter**

A general linear combination of data channels is implemented by defining a matrix of weights that is multiplied to each time sample of potentials (vector). This allowed implementation of different spatial filters, such for instance the estimation of cortical current density waveforms on the cortical ROIs.

**Spectral feature extraction**

It was performed every 40 milliseconds, using the latest 300 milliseconds of data. An autoregressive spectral estimator based on the maximum entropy algorithm yielded an amplitude spectrum with resolution of 2 Hz. Maximum frequency was limited to 60 Hz.

**Feature selection and combination**

A small subset of those spectral features (frequency bins × EEG channels or ROIs) that were significantly modulated by the motor imagery tasks was linearly combined to form a single control signal. Selection of responsive channels and frequency bins and determination of combination weights were operated before each online session (see Section 2.7). In general, only two or three spectral amplitude values (depending on individual patterns) were generally used to obtain the control signal.

**Normalization**

The control channel was detrended to avoid biases of the cursor and scaled so that the resulting vertical deflection of the feedback cursor was visible but not saturated. In fact, the vertical position of the cursor was updated every 40 milliseconds by a number of pixels (positive or negative) equal to the output by this stage. Normalization was adaptive and based on the estimate of the moving average and standard deviation of the control signal. During the very first session of each subject (screening session), since no offline analysis was available to guide feature selection and combination, the subject was given no online feedback (targets only).

### 2.7. Offline analysis

After artifact rejection, the EEG interval corresponding to the feedback phase were binned into two classes—up or down, depending on the target appeared in each trial. The spatial filtering and feature extraction stages of the online processing were replicated. Since no feedback delay issue had to be considered during the offline analysis, spectral estimation was computed on 1-second long epochs, overlapped by 50% (i.e., only five spectral estimates had to be computed for each 3-second long trial yielding about 600 spectral estimates per class for the whole session).

For each of the EEG channels or ROIs waveforms employed and for each one of the 30 frequency bins in which the EEG spectral interval was divided, a contrast was performed to assess statistically significant modulations induced on a specific feature. To this aim, we computed for each feature (dependent variable) the coefficient of determination *r* ^2^ that is the proportion of the total variance of the feature samples accounted for by target position. This index had been previously utilized in literature for similar experimental setups [11] and allows direct comparison with published results. A fictitious independent variable was created, using values +1 or −1 in correspondence of “down” or “up” epochs, respectively. A negative sign was attributed to the *r* ^2^ value when dependent and independent variables were controvariant. Viewing statistical results from a different point of view, features characterized by a high *r* ^2^ value are those that maximize prediction of the current target. Higher values of *r* ^2^ indicate that the subject has gained steadier control of EEG rhythms (in fact they generally increase during the training, from values below 0.1 to values above 0.3).

## 3. RESULTS

By applying the mentioned signal processing techniques in the context of the proposed BCI setup, we used the *r* ^2^ as an index of reliability of the recognition of subject's mental activity. The comparisons between the maximum values of the *r* ^2^that takes into account the best usable feature (frequency/ROI or scalp channel) were performed for the unprocessed EEG data as well as for the estimated cortical activity by using the procedure already described above. Mean r^2^is 0.20 ± 0.114 SD for the unprocessed EEG case, 0.55 ± 0.16 SD for the cortical current density estimation case. The differences are relatively constant across the subjects, and a paired student's t test returned a highly significant difference between the two conditions (P < 10^−5^). Once all the normals have completed the training, we choose the two with the best performance and we train them to use a different BCI application, namely, the old game of electronic ping-pong.

**Figure 2:**
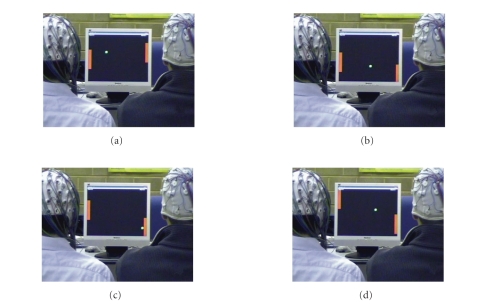
Sequence of two healthy subjects that play the ping-pong with the use of the BCI described in the text. Subjects control the cursor movement along the vertical directions. Sequence from (a) to (d).

Figure 2 shows a sequence with two subjects that played a ping-pong game with the use of the BCI system realized along the guidelines provided above. The subjects are able to control the movement of the vertical cursors while the white cursor, simulating the ball, moves across the screen. The sequence reads from (a) to (d). The two subjects are able to control the device by performing the 95 and 96% of successful hits during a game lasting several minutes, with a speed of about 5 correct hits per minute per subject.

### 3.1. Experimentation with the patients

**Figure 3:**
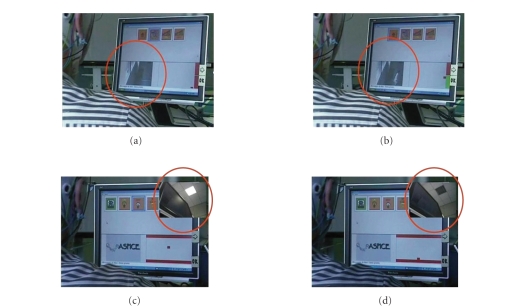
Two sequences of commands realized through the BCI systems at the foundation Santa Lucia in Rome. In the first row, foundations (a) and (b), there is a sequence with the BCI system that opens a door. In the red circles of the first row, a person enters through a door that was opened with the use of the BCI based on the EEG *μ* rhythm. The second row, (c) and (d), shows the closure of a light with the use of the same BCI system. The BCI system is controlled with the cursor at the right of the screen.

As described previously in the methods section, all the patients underwent a standard BCI training. Over the 8–12 sessions of training, four out six patients were able to develop a sensorimotor reactivity sufficiently stable to control the cursor with performance as high as over 63%. They could image either foot or hand movements and the related sensorimotor modulation was mainly located at midline centroparietal electrode positions. Two patients were not able to control the cursor with a percentage superior to 55% and were not taken into consideration further here in the context of the use of BCI system. At the end of the training, the four patients were able to control the several system outputs, namely the domotic appliances. According to the early results of the questionnaire, these patients were independent in the use of the system at the end of the training and they experienced (as they reported) “the possibility to interact with the environment by myself.” A schematic evaluation of the degree of the system acceptance revealed that amongst the several system outputs, the front door opener was the most accepted controlled device. Such application that controls the access to the domotic environment in the three-room facility rehabilitation laboratory is illustrated in the first row of Figure 3. In particular, the figure shows two sequences of commands realized through the BCI system. In the first row, (a) and (b), there is a sequence in which the BCI system was able to open a door. The red circles of the first row highlight a person that enters through the door that was opened by the successful modulation of the EEG *μ* rhythm. The second row, (c) and (d), shows the closure of a light with the use of the same BCI system. The feedback from the BCI system is displayed on the screen with the position of the cursor at the lower right of the screen.

## 4. DISCUSSION

The data reported here suggest that it is possible to retrieve the cortical activity related to the mental imagery by using sophisticated high-resolution EEG techniques, obtained by solving the linear inverse problem with the use of realistic head models. Of course, the analysis of the distribution of the potential fields associated to the motor imagery in humans has been already described [4–6, 11]. However, in the context of the brain-computer interface, it assumes importance if the activity related to the imagination of arm movement could be better detected by using such high-resolution EEG techniques than with the use of the unprocessed EEG. It is worth to note that the cortical estimation methodology illustrated above is suitable for the online applications needed for the BCI device. In fact, despite the use of sophisticated realistic head models for scalp, skull, dura mater, and cortical surface, the estimation of the instantaneous cortical distribution from the acquired potential measures required a limited amount of time necessary for a matrix multiplication. Such multiplication occurs between the data vector gathered and the pseudoinverse matrix that is stored offline before the start of the EEG acquisition process. In the pseudoinverse matrix is enclosed with the complexity of the geometrical head modeling with the boundary element or with the finite element modeling techniques, as well as the priori constraints used for the minimum norm solutions.

The described methodologies were applied in the context of the neurorehabilitation in a group of six patients affected by the Duchenne muscular dystrophy. Four out of six were also able to control with the BCI system several electronic devices disposed in a three-room facility, we described previously. The devices guided by them with an average percentage score of 63% are as follows: (i) a simple TV remote commander, with the capabilities to switch on and off the device as well as the capability to change a TV channel; (ii) the opening and closing of the light in a room; (iii) the switch on and off of a mechanical engine for opening a door of the room. These devices can be, of course, also controlled with different inputs signals that eventually uses the residual degree of muscular control of such patients. This experiment was here reported because it demonstrates the capability for the patient to accept and adapt themselves to the use of the new technology for the control of their domestic environment.

There is a large trend in the modern neuroscience field to move toward invasive electrodes implants for the recording of cortical activity in both animals and humans for the realization of an efficient BCI device [12–14]. In this paper, we have presented evidences that suggest an alternative methodology for the estimation of such cortical activity in a noninvasive way, by using the possibilities offered by an accurate modeling of the principal head structures involved in the transmission of the cortical potential from the brain surface to the scalp electrodes.
